# Raised intraocular pressure following Intravitreal Triamcinolone Acetonide in diabetic versus non-diabetic patients

**DOI:** 10.12669/pjms.345.13174

**Published:** 2018

**Authors:** Pir Salim Mahar, Abdul Sami Memon, Muhammad Faisal Fahim

**Affiliations:** 1 Prof. Dr. Pir Salim Mahar, FRCS, DO, FRCOphth. Department of Ophthalmology, Isra Ophthalmic Research & Development Center, Al-Ibrahim Eye Hospital, Isra postgraduate Institute of Ophthalmology, Karachi, Pakistan; 2 Dr. Abdul Sami Memon, FCPS. Department of Ophthalmology, Isra Ophthalmic Research & Development Center, Al-Ibrahim Eye Hospital, Isra postgraduate Institute of Ophthalmology, Karachi, Pakistan; 3 Mr. Muhammad Faisal Fahim, M.Sc. Isra Ophthalmic Research & Development Center, Al-Ibrahim Eye Hospital, Isra postgraduate Institute of Ophthalmology, Karachi, Pakistan

**Keywords:** Diabetes mellitus, Intravitreal triamcinolone acetonide, Intraocular pressure

## Abstract

**Objective:**

To determine the frequency of an increase in intraocular pressure (IOP) after intravitreal triamcinolone acetonide (IVTA) in diabetic versus non-diabetic patients with various chorio-retinal disorders.

**Methods:**

This prospective, interventional comparative case series was conducted at Isra Postgraduate Institute of Ophthalmology, Al-Ibrahim Eye Hospital, Karachi from May 2012 to April 2014. Two hundred thirty seven eyes of 180 patients were enrolled with 90 patients each in diabetic and non-diabetic group, requiring IVTA. IVTA 4mg/0.1ml was injected and IOP was measured at one week, one month, three months and six months in both groups of patients.

**Results:**

In diabetic group, 43 patients were male (47.8%) and 47 were female (52.2%), while in non-diabetic group, 56 (62.2%) patients were male and 34 (37.8%) were female. Mean age of patients in diabetic group was 52.21 ± 9.6 years and in non-diabetic group was 51.13 ± 10.75 years. The mean preoperative IOP was 13.6 ± 2.8 mmHg and 14.1 ± 2.4 mmHg in diabetic and non-diabetic group respectively. In diabetic group, mean (±SD) IOP was 16.4 ±4.9 mmHg, 14.6 ±3.6 mmHg, 17.6 ± 9.7 mmHg and 15.5 ± 7.09 mmHg at one week, one month, three months and 6 months post injection. While in non-diabetic cases, mean (±SD) IOP was 14.8 ± 3.33 mmHg, 15.9 ± 4.2 mmHg, 15.5 ± 4.2 mmHg and 14.1 ± 3.2 mmHg at one week, one month, three months and 6 months follow up. The raised IOP was observed in 117 eyes (49%) in both groups of patients, with 78 eyes (65%) in diabetic group and 39 eyes (33%) in non-diabetic group.

**Conclusions:**

After IVTA, an IOP rise was observed more in diabetics than non-diabetic patients.

## INTRODUCTION

A number of studies^1^-^5^ have reported that, individuals with diabetes mellitus (DM) experience a high prevalence of increased intraocular pressure (IOP) and primary open-angle glaucoma (POAG). However, a common link in the pathogenesis of DM and POAG has not been established. The exact mechanism of elevated IOP in POAG is not known, but an increased resistance in the outflow channels is believed for diminished outflow of aqueous humor. Rohen^6^ examined 400 trabeculectomy specimens of glaucomatous eyes with ultra-structural analysis and found, three types of extracellular deposits containing glycoproteins within the cribriform layers of the trabecular meshwork. The presence of fibronectin, an extracellular glycoprotein in the trabecular tissue, mainly, in the inner wall of schlemm’s canal and juxtacanalicular, or cribriform part of trabecular meshwork has been verified by other workers also.^7^ Glucose concentration is believed to be higher in aqueous humor of diabetic patients. Davies^8^ measured glucose concentration in aqueous of 56 patients undergoing cataract surgery. The mean aqueous glucose level was 3.2 millimolar (mM) in non-diabetics compared to 7.8mM in diabetic patients. Several studies have now recognized that increased glucose level in aqueous induces increase in fibronectin synthesis and its accumulation in the trabecular meshwork with depletion of normal trabecular meshwork cells.^9^,^10^ Is fibronectin synthesis in the trabecular meshwork a missing link for increase prevalence of glaucoma in DM has to be proven in further studies.

Triamcinolone acetonide, a synthetic glucocorticoid is used intravitrealy to treat a variety of ocular diseases such as cystoid macular edema (CME) after cataract surgery,^11^ macular edema due to retinal vascular conditions, such as diabetic retinopathy,^12^ central retinal vein occlusion (CRVO),^13^ branch retinal vein occlusion (BRVO)^14^ and macular edema due to inflammatory conditions e.g. uveitis and birdshot retinochoroidopathy.^15^ Most common complication of intravitreal triamcinolone acetonide (IVTA) reported in literature is raised IOP.^16^,^17^ We have been using IVTA our patients several years in patients with retinal and choroidal vascular disorders and our clinical impression has been that we were witnessing an increase IOP more in diabetic than non-diabetic patients.

To prove the hypothesis that diabetics are more prone to elevated IOP than non-diabetic patients, we undertook this study to determine the frequency of IOP elevation following IVTA in diabetic versus non-diabetic patients having various retinal pathology with a final follow up of six months.

## METHODS

This prospective, interventional comparative case series study was conducted at Isra Postgraduate Institute of Ophthalmology, Al-Ibrahim Eye Hospital, Karachi from May 2012 to April 2014. The permission of study was granted by the hospital’s ethics committee and was performed in accordance with Declaration of Helsinki. A non-probability purposive sampling technique was used for data collection. Sample size was calculated from the online software www.raosoft.com by taking the 5% margin of error, 95% confidence interval and estimated sample size was drawn to be 180. All patients provided informed consent. Two hundred thirty seven eyes of 180 patients were enrolled in the study with 90 patients (119 eyes) in diabetic and 90 patients (118 eyes) in non-diabetic group, requiring IVTA injection. Patients having IOP of more than 21 mmHg, receiving anti-glaucoma medication or history of glaucoma surgery and family history of glaucoma were excluded from the study. Patients who received IVTA previously within six months of the study and patients receiving any anti-vascular endothelial growth factor (VEGF) intravitreal injections in past within three months of the study were also excluded. All patients without any history of diabetes had two consecutive baseline random blood sugar (RBS) levels, followed by fasting blood plasma level and glycated hemoglobin (HbA1c) to avoid any recruitment bias. Pre-IVTA assessment of patients included detailed medical history with the assessment of best corrected visual acuity (BCVA), biomicroscopic examination of anterior segment with double mirror Goldmann lens gonioscopy and dilated fundus examination with 90 diopter volk lens and indirect ophthalmoscope. The ancillary investigation included fundus fluorescein angiography (FFA – Kowa VX 10i) and optical coherence tomography (OCT – Topcon 2000) of posterior segment. The baseline IOP measurement was established by taking the mean of two highest values measured at 9:00am and at 4:00pm by Goldmann applanation tonometer (GAT) to reduce any error due to diurnal variation. All data regarding patient’s examination, diagnosis, treatment and follow up were entered in pre-designed proforma.

All patients were treated with topical antibiotics (Moxifloxacin 0.5% - Vigamox, Alcon, Belgium), 24 hours prior to IVTA and continued for three days, post-injection. The intravitreal injections were given in operating theatre under sterile condition with preparing of the eyes with 5% povidone-iodine and delivering triamcinolone acetonide (Kenacort – Ophth Lab, Karachi, Pak) in a dose of 4mg/0.1ml through pars plana into vitreous cavity, under topical anesthesia (Alcaine – Alcon, Belgium).

Patients were followed at one week, one month, three months and six months subsequently. At each visit, patients had detailed ocular examination with measurement of IOP. If IOP was found to be more than 21 mmHg.

### Statistical Analysis

Statistical Package for Social Sciences (SPSS) version 20.0 was used for data analysis. Frequency and percentages were computed for categorical variables including gender and diagnosis. Mean ± standard deviation was calculated for IOP and age. Independent sample t-test was used to compare the significance of mean IOP between diabetic and non-diabetic group and paired sample t-test was applied at different follow-ups (one week, one month, three months and 6 months). P-value < 0.05 considered to be statistically significant.

## RESULTS

A total number of 237 eyes (180 patients) were included in the study with 90 patients each in diabetic and non-diabetic group. In diabetic group, 43 patients were male (47.8%) and 47 were female (52.2%), while in non-diabetic group, 56 (62.2%) patients were male and 34 (37.8%) were female. Mean age of patients in diabetic group was 52.21 ± 9.6 years and in non-diabetic group was 51.13 ± 10.75 years. The distribution of patients according to their diagnosis is shown in Table-I. Diabetic macular edema was the most frequent diagnosis in diabetic (n = 68 patients) while BRVO was in non-diabetic cases (n = 31).

**Table-I:** Distribution of patients according to the diagnosis (n = 180).

Diagnosis	Diabetic n = 90 Cases	Non-Diabetic n = 90 Cases
DME	68 (75.6%)	0
Neovascular ARMD	2 (2.2%)	10 (11.1%)
BRVO	9 (10%)	31 (34.4%)
CRVO	11 (12.2%)	25 (27.8%)
Uveitis	0	24 (26.7%)

Data shown in frequencies and percentages n (%) **DME:** Diabetic Macular Edema, **ARMD:** Age related macular degeneration, **BRVO:** Branch retinal vein occlusion, **CRVO:** Central retinal vein occlusion.

The mean preoperative IOP was 13.6 ± 2.8 mmHg and 14.1 ± 2.4 mmHg in diabetic and non-diabetic group respectively. The difference between preoperative mean IOPs in both groups was insignificant (p-value = 0.287).

In diabetic group, mean (±SD) IOP was 16.4 ±4.9 mmHg, 14.6 ± 3.6 mmHg and 17.6 ± 9.7 mmHg, 15.5 ± 7.09 mmHg at one week, one month, three months and six months post injection. While in non-diabetic eyes, mean (±SD) IOP was 14.8 ± 3.33 mmHg, 15.9 ± 4.2 mmHg, 15.5 ±4.2 mmHg and 14.1 ± 3.2 mmHg at one week, one month, three months and six months follow up (Table-II & Fig.1). At one week and three month follow up, the mean IOP was significantly high in diabetic group with, p-value = 0.003 and p-value = 0.029 respectively while at one month follow up, mean IOP was significantly high in non-diabetic group with p-value = 0.012. A rise in IOP to value higher than 21 mmHg was observed in 21 (17.6%) eyes and seven (5.9%) eyes in diabetic and non-diabetic group respectively after one week of IVTA. After one month, raised IOP was observed in 4 (3.4%) and 17 (14.4%) eyes in diabetic and non-diabetic group. At one month follow up, IOP was significantly high in non-diabetic group. At 3 months follow-up, a rise in IOP was observed in 28 (23.5%) and 11 (9.3%) eyes in diabetic and non-diabetic group and at 6 months, high IOP was witnessed in 25 (21%) eyes and 4 (3.4%) eyes in diabetic and non-diabetic group respectively (Table-III).

**Table-II:** Mean IOP after IVTA at four different follow ups.

Post-Op IOP (mmHg)	Diabetic (n = 119 eyes)	Non-Diabetic (n = 118 eyes)	P-Values
1 Week	16.4 ± 4.9 (10 – 30)	14.8 ± 3.33 (10 – 26)	0.003
1 Month	14.6 ± 3.6 (10 – 32)	15.9 ± 4.2 (10 – 30)	0.012
3 Months	17.7 ± 9.7 (10 – 48)	15.5 ± 4.0 (10 – 30)	0.029
6 Months	15.5 ± 7.09 (10 – 42)	14.1 ± 3.2 (10 – 27)	0.047

**IOP:** Intraocular pressure; **IVTA:** Intravitreal triamcinolone acetonide P-value < 0.05 considered statistically significant All p-values were measured using Independent sample t-test Data shown is mean IOP ± SD and range as minimum to maximum

**Table-III:** Frequency of raised IOP in eyes.

Follow ups	Raised IOP (IOP > 21 mmHg)	

	Diabetic (n = 119 eyes)	Non Diabetic (n = 118 eyes)
1 Week	21 (17.6%)	7 (5.9%)
1 Month	4 (3.4%)	17 (14.4%)
3 Months	28 (23.5%)	11 (9.3%)
6 Months	25 (21%)	4 (3.4%)

Data shown in frequencies and percentages n (%) **IOP:** Intraocular pressure.

Our results showed that out of 237 eyes enrolled in the study, 117 eyes (49%) showed IOP increase above 21mmHg. Out of 117 eyes, 78 eyes (65%) were in diabetic patients compared to 39 eyes (33%) in non-diabetic patients

## DISCUSSION

The raised IOP is one of the major unwanted outcomes of IVTA. In a mixed population locally, Mahar and Memon^17^ witnessed an increase in IOP in 38% of eyes. There are several mechanisms proposed for corticosteroids to cause an increase in IOP. Corticosteroids increase expression of extracellular matrix proteins, fibronectin, polymerised glycosaminoglycans and elastin with their accumulation in the trabecular meshwork, obstructing the outflow pathway.^18^ The endothelial cells of trabecular meshwork are phagocytic, removing debris from meshwork. Corticosteroids are known to suppress the phagocytic activity of these cells, allowing extra debris to accumulate in the trabecular meshwork.^19^ Crystalline deposits of triamcinolone acetonide are also believed in causing physical obstruction of the trabecular meshwork.^20^ Corticosteroids are also alleged to cause glucocorticoid receptor-mediated cross linkage of actin-filament network^21^ and an expression of protein myocilin in trabecular meshwork impeding aqueous outflow.^22^

Patients with diabetic mellitus are more prone to have higher IOP and increased prevalence of POAG and ocular hypertension. The higher IOP in black population in Barbados Eye Study^1^ was linked to the higher prevalence of DM. The presence of DM was associated with an overall rise in mean IOP of patients in Rotterdam Study also.^2^ Katz and Sommer^3^ examined 94 individuals with POAG having well documented glaucomatous visual field loss, compared to similar number of controls, matched by age and gender. DM showed the closest association with glaucoma. The sub-group analysis for whites and blacks people showed DM to be a risk factor for both groups. The Blue Mountain Eye Study^4^ showed that, glaucoma prevalence was increased in people with DM, diagnosed from history or increased fasting plasma glucose level (5.5%) compared with those without DM (2.8%). Ocular hypertension was also more common in diabetic patients (6.7%), compared with those without DM (3.5%). Overall, DM was present in 13% of people with glaucoma, compared with 6.9% of those, without glaucoma. This study also determined that, patients, not receiving any anti-glaucoma therapy showed higher IOP at presentation. Klein and coworkers^5^ measured IOP in 2366 diabetic persons and 381 non-diabetics and found higher mean IOP in persons with DM. In this study, a positive history of glaucoma was also higher in diabetic population. Their study suggested an increased risk of glaucoma, when evaluating a diabetic patient.

An increase in IOP is a recognized complication of IVTA. In our study we divided patients in diabetic and non-diabetic group to see if patients with history of DM are more prone to have elevated IOP than their non-diabetic counterparts after IVTA. As intravitreal corticosteroids will be continuously used for various retinal and choroidal vascular disorders, one has to be careful in monitoring IOP for six months post-injection, especially in patients with history of DM. The limitation of our study is that we fixed the eye as our variable and also we defined an increase in IOP above 21 mmHg.

## CONCLUSION

There was a higher incidence of raised IOP (>21 mmHg) among diabetic patients compared with non-diabetics after IVTA. We feel that diabetic patients should be closely monitored for raised IOP after IVTA and if possible alternate drugs should be considered for Intravitreal use.

### Authors’ Contribution

**PSM** conceived, designed, writing of manuscript and takes all the responsibility.

**ASM** did data collection did review of manuscript.

**MFF** did statistical analysis, editing & reviewing of manuscript.
